# Do Sharpness-Based Optimizers Improve Generalization in Medical Image Analysis?

**DOI:** 10.1109/ACCESS.2025.3568641

**Published:** 2025-05-09

**Authors:** MOHAMED HASSAN, ALEKSANDAR VAKANSKI, BOYU ZHANG, MIN XIAN

**Affiliations:** Department of Computer Science, University of Idaho, Idaho Falls, ID 83402, USA

**Keywords:** Deep learning, generalization, medical image analysis, loss landscape, sharpness-aware minimization

## Abstract

Effective clinical deployment of deep learning models in healthcare demands high generalization performance to ensure accurate diagnosis and treatment planning. In recent years, significant research has focused on improving the generalization of deep learning models by regularizing the sharpness of the loss landscape. Among the optimization approaches that explicitly minimize sharpness, Sharpness-Aware Minimization (SAM) has shown potential in enhancing generalization performance on general domain image datasets. This success has led to the development of several advanced sharpness-based algorithms aimed at addressing the limitations of SAM, such as Adaptive SAM, Surrogate-Gap SAM, Weighted SAM, and Curvature Regularized SAM. These sharpness-based optimizers have shown improvements in model generalization compared to conventional stochastic gradient descent optimizers and their variants on general domain image datasets, but they have not been thoroughly evaluated on medical images. This work provides a review of recent sharpness-based methods for improving the generalization of deep learning networks and evaluates the methods’ performance on three medical image datasets, including breast ultrasound, chest X-ray, and colon histopathology images. Our findings indicate that the initial SAM method successfully enhances the generalization of various deep learning models. While Adaptive SAM improves generalization of convolutional neural networks, it fails to do so for vision transformers. Other sharpness-based optimizers, however, do not demonstrate consistent results. The results reveal that contrary to findings in the non-medical domain, SAM is the only recommended sharpness-based optimizer that consistently improves generalization in medical image analysis, and further research is necessary to refine the variants of SAM to enhance generalization performance in this field.

## I. INTRODUCTION

Deep neural networks (DNNs) have demonstrated success across different tasks in recent years, such as computer vision, natural language processing, and video/speech recognition [1]. However, these networks are typically over-parametrized and are prone to overfitting the training data, resulting in discrepancy in the generalization performance on unseen data [2]. Improving the generalization performance is crucial, as a model’s ability to perform well on unseen data is arguably the most important factor determining the algorithm’s usability in real-life applications. Generalization poses a greater challenge in medical image analysis, as medical image datasets differ from non-medical image datasets in several ways: they typically contain fewer images, have higher resolution, lower contrast, and a lower signal-to-noise ratio [3]. Additionally, medical images are collected using various devices, protocols, and patient populations, introducing further complexities that hinder generalization on unseen images.

To understand the generalization phenomenon of DNNs, prior studies have developed complexity measures that correlate monotonically with generalization performance. Complexity measures have been proposed based on statistical learning theory bounds and/or empirical studies based on the network size [4], [5], the norm of the model parameters [6], [7], the geometry of the loss landscape [8], and other factors. Jiang et al. [9] evaluated 40 measures using the Network-in-Network (NiN) architecture on the CIFAR-10 and SVHN datasets using Kendall’s rank correlation coefficient. Similarly, Dziugaite et al. [10] evaluated 24 complexity measures on CIFAR-10 and SVHN datasets by investigating the causal relation between various measures and generalization using conditional independence tests. Both studies concluded that many norm-based and network size measures performed poorly and stated the potential of PAC-Bayes sharpness-based measures for establishing a connection between the geometry of the loss landscape and generalization. Motivated by this, Vakanski and Xian [3] conducted a correlation experiment akin to Jiang et al. [9] on medical images, assessing the correlation of 25 different complexity measures with generalization using a VGG-like network. The authors reported that PAC-Bayes sharpness measures exhibited the highest positive correlation with generalization on medical images.

The connection between the geometry of the loss landscape and generalization has received a good amount of research as most state-of-the-art overparametrized DNNs, including convolutional neural networks (CNNs) and vision transformers, possess complex landscapes with numerous sharp minima [11]. In such an overparametrized setting, although solutions with sharp minima minimize the training error, many of them do not generalize well [12]. Chaudhari et al. [13] search for flat regions by minimizing the local entropy using an approach suitably called Entropy-SGD. Wen et al. [14] introduced a SmoothOut technique that focuses on smoothening out the sharp minima for higher generalization. Lewkowycz et al. [15] claim that SGD with large learning rates generalizes well since dynamical instability at initialization causes the optimizer to catapult to regions where the maximum eigenvalue of the neural tangent kernel and of the Hessian are smaller. Motivated by previous work, Foret et al. [16] propose Sharpness Aware Minimization (SAM) as a learning algorithm based on the PAC-Bayesian generalization bound that improves model generalization performance on various natural image and language benchmarks by simultaneously minimizing both training loss and sharpness. SAM seeks flat minima by employing adversarial perturbations to maximize the training loss and then minimizing the loss of this perturbed objective through a single update step of a base optimizer, such as the Adam optimizer. However, SAM has limitations in relation to sensitivity to parameter rescaling and the non-linearity of the loss landscape. These limitations, despite SAM’s strong performance, have inspired the development of new sharpness-based optimization algorithms, including Adaptive Sharpness-Aware Minimization (ASAM) [17], Surrogate Gap Guided Sharpness-Aware Minimization (GSAM) [18], Weighted Sharpness-Aware Minimization (WSAM) [19], and Curvature Regularized Sharpness-Aware Minimization (CR-SAM) [20].

Our work was inspired by previous studies that evaluated the generalization performance of SAM and its variants on non-medical benchmark datasets (e.g., CIFAR-10/100 and ImageNet) using CNNs [16], [17], [18], [19], [20], [21] and large language models (LLMs) [22]. These studies demonstrated improvements in model generalization compared to Adam optimizers for all sharpness-based optimizers, including SAM, ASAM, GSAM, WSAM, and CR-SAM. However, prior works have not thoroughly evaluated the effectiveness of sharpness-based models on medical images or with vision transformer models. Additionally, some of the related work focused solely on evaluating generalization performance without validating the geometry of the loss landscape [21], [22].

In this paper, we first survey the relevant recent sharpness-based methods for improving the generalization of neural networks. Next, we assess sharpness-based algorithms on medical breast ultrasound (BUS) images and compare their performance to baseline models using the Adam optimizer. Our objective is to investigate whether SAM approaches enhance the generalization performance of medical images. Toward this goal, we evaluate the performance of classification models, which include two popular CNN-based models, ResNet50 [23] and VGG16 [24], and two Vision Transformer models, ViT [25] and Swin Transformer [26]. In addition to BUS images, we extend our analysis to cross-domain generalization on the BUSIS dataset and further evaluate on two other large open-access medical datasets: CheXpert and PathMNIST. We utilize performance metrics derived from the approximation of the Hessian matrix to quantify the connections between the geometry of the loss landscape and generalization. We also report the computational costs of sharpness-based methods to determine their efficiency and scalability for deployment in real-life applications. Our results indicate that SAM is the only sharpness-based optimizer that consistently enhances the generalization performance of all tested models. Additionally, Hessian-based metrics demonstrate a flatter landscape produced by SAM in comparison to the standard Adam optimizer for all tested models. In contrast, ASAM, GSAM, WSAM, and CR-SAM did not exhibit consistent improvements across our experiments. Furthermore, our analysis of computational costs highlights the efficiency of SAM and CR-SAM, which exhibit significantly lower computational overhead compared to ASAM, GSAM, and WSAM, making them more suitable for deployment in real-life applications. Our results reveal that SAM has the highest potential among all sharpness-based optimizers in the domain of medical image analysis.

The main contributions of this work are as follows:

- Overviews and provides a comparative study of the performance of the most common sharpness-based algorithms: SAM, ASAM, GSAM, WSAM, and CR-SAM.
- Evaluates the generalization performance of sharpness-based algorithms on medical BUS images by utilizing Hessian metrics to investigate changes in the geometry of the loss landscape.
- Extends the evaluation to cross-domain generalization on the BUSIS dataset and additional open-access medical datasets (CheXpert and PathMNIST), comparing the performance of sharpness-based algorithms across CNN-based and Vision Transformer models.

## II. METHOD

This section first provides an overview of sharpness-based optimizers, including SAM and its variants. Afterward, it presents the metrics used in investigating the geometry of the loss landscape.

### A. SHARPNESS AWARE MINIMIZATION (SAM)

The geometry of the loss landscape can influence how well a model generalizes on new datasets. Keskar et al. [8] suggested that models with a flat loss landscape (flat minimum) generalize better than models with a sharp loss landscape (sharp minimum), as the large sensitivity of the training function at a sharp minimizer negatively impacts the trained model’s ability to generalize on new data. The generalization gap of DNNs can be formally defined as the difference between the training and test losses. Mathematically, it is defined by:

(1) $$\text{Generalization gap :}\;L_{D}(w)-L_{S}(w),$$

where w is the weight vector, $L_{S}(w)$ is the training loss, $L_{D}(w)$ is the generalization loss. The connection between the generalization gap and sharpness of the loss function within an ϵ-ball is satisfied according to the following definition:

(2) $$\text{Sharpness :}\;\underset{\|\epsilon \|p\leq \rho }{\max}L_{S}(w+\epsilon )-L_{S}(w)\text{,}$$

where ρ is the radius of the maximization region which is an ${\text{𝓵}}^{p}$ ball. From the above definition, the sharpness function is the difference between the maximum training loss in the ${\text{𝓵}}^{p}$ ball with a fixed radius ρ and the training loss.

Motivated by this work, Foret et al. [16] propose a learning algorithm called Sharpness Aware Minimization (SAM) that combines the geometry of the loss landscape and a PAC-Bayes norm as a generalization bound. SAM focuses on minimizing the sharpness of the loss landscape during training to seek a flat minima by minimizing the following PAC-Bayesian generalization error bound:

(3) $$L_{D}(w)\underset{\|\epsilon \|p\leq \rho }{max}\leq L_{S}(w+\epsilon )+h\left(\frac{\|w{\|}_{2}^{2}}{\rho^{2}}\right),$$

for a strictly increasing function h. Because of the monotonicity of h, it can be substituted by ${\text{𝓵}}^{2}$ weight decaying regularizer. Hence, SAM can be defined as the following minimax optimization:

(4) $$\underset{w}{\min}\underset{\|\epsilon \|p\leq \rho }{\max}L_{S}(w+\epsilon )+\frac{\lambda }{2}\|w{\|}_{2}^{2},$$

where λ is a weight decay coefficient.

### B. ADAPTIVE SHARPNESS AWARE MINIMIZATION (ASAM)

Kwon et al. [17] claim that some sharpness-based learning methods, including SAM, suffer from sensitivity to model parameter rescaling, which may weaken the correlation between sharpness and the generalization gap. Sharpness defined in the rigid spherical region with a fixed radius has a weak correlation with the generalization gap because of the non-identifiability of neural networks with ReLU activation functions, whose parameters can be freely rescaled without affecting its output [27]. The scale-dependency can cause weaker correlation between sharpness and generalization. This can be represented by the following equation:

(5) $$\underset{\|\epsilon {\|}_{2}\leq \rho }{\max}L_{S}(w+\epsilon )\neq \underset{\|\text{ϵ}{\|}_{2}\leq \rho }{\max}L_{S}(Aw+\epsilon ),$$

where A is a scaling operator. Kwon et al. eliminate the vulnerability to weight scaling, by introducing a normalization operator. Considering that $\left\{T_{w},w\in {\mathbb{R}}^{k}\right\}$ is a family of invertible linear operators on ${\mathbb{R}}^{k}$, if $T_{Aw}^{-1}A=T_{w}^{-1}$ for any invertible scaling operator A on ${\mathbb{R}}^{k}$ which does not change the loss function, then $T_{w}^{-1}$ is the normalization operator of w. Using the normalization operator, Kwon et al. define adaptive sharpness as follows:

(6) $$\underset{\left\|T_{w}^{-1}\epsilon \right\|p\leq \rho }{max}L_{S}(w+\epsilon )-L_{S}(w)$$

The concept of adaptive sharpness is used to formulate Adaptive Sharpness-Aware Minimization (ASAM) as follows:

(7) $$\underset{w}{min}\underset{\left\|T_{w}^{-1}\epsilon \right\|p\leq \rho }{max}L_{S}(w+\epsilon )+\frac{\lambda }{2}\|w{\|}_{2}^{2}$$

ASAM induces minimization of the generalization loss without the sensitivity to weight scaling, and due to the negligible calculation cost of $T_{w}^{-1}$, ASAM can be as computationally efficient as SAM.

### C. SURROGATE GAP GUIDED SHARPNESS-AWARE MINIMIZATION (GSAM)

Zhuang et al. [18] argue that both sharp and flat minima can exhibit a low perturbed loss, implying that SAM doesn’t always prefer flat minima. Motivated by this limitation, Zhuang et al. propose a surrogate gap, a measure equivalent to the dominant eigenvalue of the Hessian matrix at a local minimum when the radius of neighborhood is small. The surrogate gap can hence serve as an equivalent measure of curvature at minima, and it is defined by:

(8) $$h(w)≜L_{p}(w)-L(w),$$

where $L_{p}(w)≜\underset{\|\delta \|\leq p_{t}}{\max}L\left(w_{t}+\delta \right)$ returns the worst possible loss within a ball of radius $p_{t}$ centered at $w_{t}$, and L(w) is the loss function with parameter $w\in {\mathbb{R}}^{k}$. Using the surrogate gap, Zhuang et al. [18] propose Surrogate Gap Guided Sharpness Aware Minimization (GSAM) as follows:

(9) $$min\left(L_{p}(w),h(w)\right),$$

where $L_{p}(w)$ minimizes the training loss, and h(w) minimizes the generalization gap. GSAM consists of two steps; a gradient descent $\nabla L_{p}(w)$ similar to SAM to minimize the perturbed loss $L_{p}(w)$, and an ascent step orthogonal to $\nabla L_{p}(w)$ to minimize the surrogate gap without affecting the perturbed loss.

### D. WEIGHTED SHARPNESS-AWARE MINIMIZATION (WSAM)

Yue et al. [19] claim that SAM finds flatter regions but not minima, which could potentially lead to convergence at a point where the loss is still large. This motivated the authors to propose a more general method, called WSAM, by introducing sharpness as a regularization term.

(10) $$L^{WSAM}(w):=L(w)+\frac{\gamma }{1-\gamma }\tilde{L}(w)\;\;=\frac{1-2\gamma }{1-\gamma }L(w)+\frac{\gamma }{1-\gamma }L^{SAM}(w),$$

In the above equation, L(w) is the loss function, $\tilde{L}(w)$ approximates the dominant eigenvalue of the Hessian at local minima, and $\gamma /(1-\gamma )\tilde{L}(w)$ is the regularized weighted sharpness term. The hyperparameter γ∈[0,1) controls the weight of the sharpness to direct the loss trajectory to find either flatter or lower minima. When γ=0, the loss term $L^{WSAM}(w)$ degenerates to a vanilla loss, when γ=1/2, $L^{WSAM}(w)$ is equivalent to the original SAM algorithm (i.e., $L^{SAM}(w)$), and when γ>1/2, the term $L^{WSAM}(w)$ gives more weights to the sharpness, so that it finds the point which has smaller curvature rather than smaller loss.

To control the regularization term, Yue et al. use a weight decouple technique inspired by Loshchilov and Hutter [28] so that it reflects the sharpness of the current step without additional information, where $\tilde{L}(w)$ is not integrated into the base optimizer to calculate the gradients and update weights, but it is calculated independently. The authors claim that WSAM is more efficient than SAM as it can achieve different minima by choosing different values of γ.

### E. CURVATURE REGULARIZED SHARPNESS-AWARE MINIMIZATION (CR-SAM)

Wu et al. [20] posit that because SAM aims to improve generalization by minimizing worst-case loss using one-step gradient ascent as an approximation, such one-step gradient ascent approach becomes less effective as the training progresses due to the non-linearity of the loss landscape. On the other hand, using a multi-step ascent gradient approach will cause higher training costs. To tackle this issue, Wu et al. propose Curvature Regularized SAM (CR-SAM) by integrating a normalized Hessian trace C(w) that measures the curvature of the loss landscape.

(11) $$C(w)=\frac{TR\left(\nabla^{2}L_{S}(w)\right)}{{\left\|\nabla L_{S}(w)\right\|}_{2}}$$

The issue with the normalized Hessian trace is that a direct minimization of C(w) would lead to an increase in the gradient norm ${\left\|\nabla L_{S}(w)\right\|}_{2}$, which could adversely affect generalization. Therefore, Wu et al. optimize $TR\left(\nabla^{2}L_{S}(w)\right)$ and ${\left\|\nabla L_{S}(w)\right\|}_{2}$ separately as shown below:

(12) $$R_{c}(w)=\alpha logTR\left(\nabla^{2}L_{S}(w)\right)+\beta \log{\left\|\nabla L_{S}(w)\right\|}_{2}$$

In the above formula, α>β>0, so that the numerator of C(w) is penalized more than the denominator. The term $R_{c}(w)$ is a combination of normalized Hessian trace with gradient norm penalty regularizer, and is used as a regularization term in SAM as shown below:

(13) $$L^{CR-SAM}(w):=L^{SAM}(w)+R_{c}(w)$$

Since computing the Hessian trace as in $R_{c}(w)$ for large matrices in over-parameterized DNNs can be computationally intensive, Wu et al. [20] propose an approximation based on finite difference (FD), which reduces the complexity in a computation parallelizable way. Wu et al. state that the parallelism in CR-SAM optimizes the worst-case and best-case perturbations within the parameter space (ρ-bounded neighborhood) promoting a smoother, flatter loss function with same training speed as SAM.

### F. HESSIAN-BASED METRICS

Model analysis by using metrics derived from the Hessian matrix is widely utilized in scientific computing. To address the loss landscape curvature information, we use the PyHessian [29] framework, which directly analyzes Hessian (second-derivative) information with respect to the model parameters. Considering a deep learning network with m parameters, the gradient of the loss with respect to model parameters is a vector:

(14) $$\frac{\partial L}{\partial \theta }=g_{\theta }\in {\mathbb{R}}^{m},$$

and the second derivative of the loss is the matrix:

(15) $$H=\frac{\partial^{2}L}{\partial \theta^{2}}=\frac{\partial g_{\theta }}{\partial \theta }\in {\mathbb{R}}^{m\times m}$$

Given that deep learning networks typically have millions or billions of parameters, forming the Hessian matrix explicitly becomes computationally unfeasible. Instead, properties of the Hessian spectrum are often computed without explicitly forming the matrix. This is achieved by using an oracle to compute the multiplication of the Hessian to a random vector v, as shown below:

(16) $$\frac{\partial g_{\theta }^{T}v}{\partial \theta }=\frac{\partial g_{\theta }^{T}}{\partial \theta }v+g_{\theta }^{T}\frac{dv}{d\theta }=\frac{\partial g_{\theta }^{T}}{\partial \theta }=Hv,$$

where the cost of this Hessian matrix-vector multiply (referred to as a Hessian matvec) is the same as one gradient backpropagation. Having this Hessian matvec, the top Hessian eigenvalue is computed using power iteration [30] to reveal the principle curvatures of the loss landscape, indicating the directions of greatest descent or ascent. Positive eigenvalues suggest an ascent toward a local minimum, while negative eigenvalues indicate a descent from a local minimum. Smaller eigenvalues (closer to 0) generally correspond to flatter regions in the loss landscape, which are associated with better generalization performance.

To obtain additional information on the geometry of the loss landscape, the trace of the Hessian can be computed using Hutchinson’s method [31] by sampling the random vector v from a Gaussian distribution with mean 0 and variance 1, resulting in the following identity:

(17) $$Tr(H)=Tr(HI)=Tr\left(HE\left[vv^{T}\right]\right)\;\;=E\left[\text{Tr}\left(\text{H}vv^{T}\right)\right]=E\left[v^{T}Hv\right],$$

where I is an identity matrix of appropriate size. The trace of the matrix Tr(H) is estimated by computing the expectation of drawing multiple random samples $E\left[v^{T}Hv\right]$, where $v^{T}Hv$ is a dot product between the Hessian matvec and the original vector v.

In addition to the top Hessian eigenvalue and the Hessian trace, we compute the median, mean and standard deviation of the Hessian eigenvalues by sampling 100 different weight perturbations for every batch in the train set. This information offers a more comprehensive understanding of the curvature of the loss landscape. While the top Hessian eigenvalue and the Hessian trace provide valuable information about the largest curvature of the loss landscape, they may not fully represent the overall shape and distribution of the curvatures. A loss landscape with low mean, median, and standard deviation values typically indicate smoother optimization paths.

## III. EXPERIMENTAL RESULTS

### A. BREAST ULTRASOUND DATASET

**FIGURE 1.**
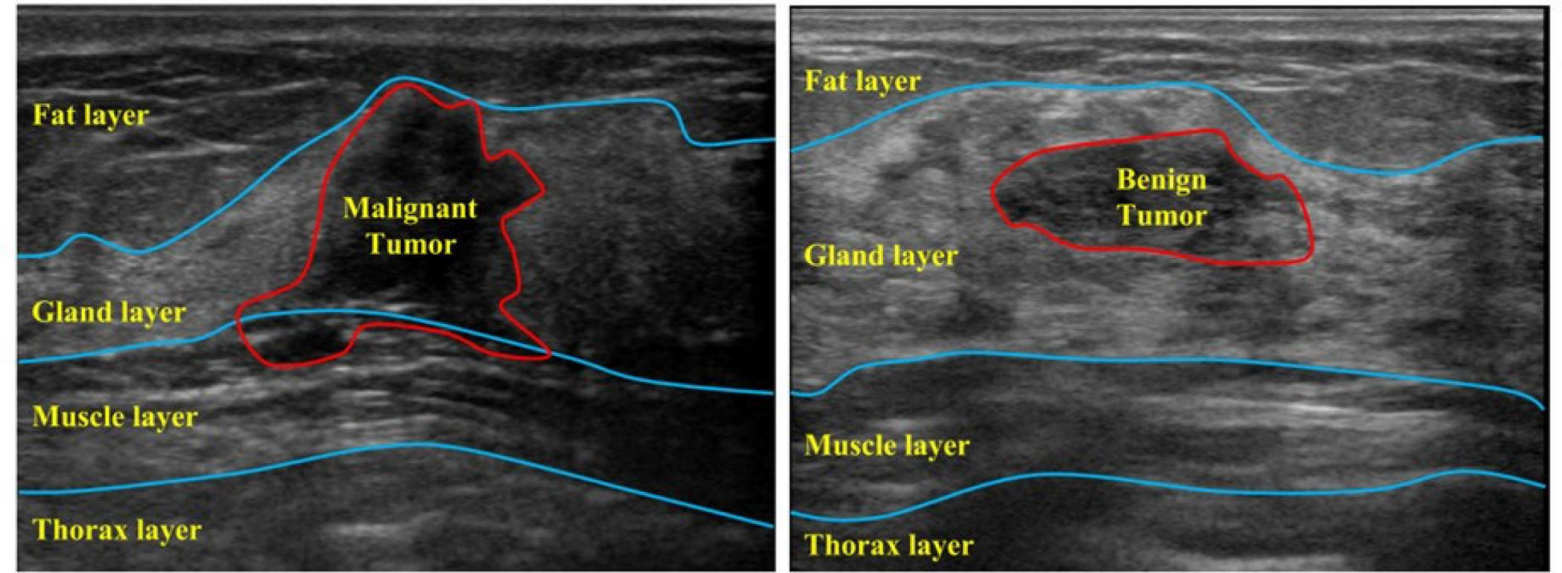
GDPH&SYSUCC Dataset sample Malignant image (left) and Benign image (right).

Breast cancer ranks as the most frequently diagnosed cancer among women and remains one of the leading causes of cancer-related deaths worldwide [32]. The primary goal in combating breast cancer is to reduce its mortality rate by identifying signs and symptoms at an early stage. Deep learning models have been applied to breast cancer diagnosis via mammography, ultrasonography, and magnetic resonance imaging [33]. However, medical images often suffer from poor image quality due to factors such as speckle noise, low contrast, weak boundary definition, and variations in tumor size and echo strength across patients [34], [35]. These challenges contribute to the limited generalization performance of deep learning models in this domain. In this paper, we use a BUS dataset, GDPH&SYSUCC, which is the largest open dataset currently in this field [36], [37]. This dataset is collected from two medical centers: the Department of Ultrasound, Guangdong Provincial People’s Hospital (GDPH) and the Department of Ultrasound, Sun Yat-sen University Cancer Center (SYSUCC). The images were acquired with the following equipment: Mindray DC-80 from China, Hitachi Ascendus and Toshiba Aplio 500 from Japan, and Supersonic Aixplorer from France. It consists of 886 benign and 1,519 malignant images, for a total of 2,405 BUS images. BUS images include four layers: fat layer, gland layer, muscle layer, and thorax layer (as shown in Fig. 1), where malignant tumors start from the gland layer and tend to invade into deeper layers vertically, while benign tumors start at the gland layer and likely to extend horizontally within the gland.

### B. EXPERIMENTS

To establish a comprehensive comparative analysis between sharpness-based optimizers and the baseline Adam optimizer, we applied these methods across a diverse set of medical imaging tasks, including Breast Ultrasound (BUS), cross-domain evaluation on the BUSIS dataset, chest X-ray (CheXpert), and colon cancer histopathology (PathMNIST). We tested all optimizers using CNN-based models such as ResNet50 and VGG16, as well as Vision Transformer architectures like ViT and Swin Transformer. Hyperparameter selection was carried out through grid search, optimizing learning rate values of $\left\{10^{-3},10^{-4},10^{-5}\right\}$, batch sizes of {8,16,64}, and sharpness-aware radius (ρ) within the range [0.01, 2.0], WSAM’s weight hyperparameter γ within the range [0.1, 0.9], and CRSAM’s coefficients α and β within the range [0.01, 0.90]. The best performing hyperparameters found via grid search were as follows and remained consistent across all experiments and datasets unless otherwise noted. For SAM, a perturbation radius ρ of 0.05 was used across all models. ASAM used a larger radius of ρ=0.5. GSAM performed best with ρ=1.0. For WSAM, the optimal configuration was ρ=0.2 with sharpness weight γ=0.8. CRSAM used ρ=0.15, α=0.10, and β=0.01 for CNN-based models, and ρ=0.10, α=0.05, and β=0.01 for Vision Transformer architectures. Models were trained from scratch until they achieved 100% training accuracy, with early stopping applied to prevent overfitting. These settings align with the experimental protocols used in similar studies by Jiang et al. [9] and Dziugaite et al. [10].

#### 1) EXPERIMENT 1: BASELINE MEASUREMENT

To establish a baseline measurement for comparing the sharpness-based optimizers, we used subsets of the train (80%) and test (20%) images, an Adam optimizer with a learning rate of 10^−4^, and a batch size of 16. We trained two Convolution Neural Network models, ResNet50 and VGG16, and two Vision Transformer models, ViT and Swin transformer, until achieving 100% training accuracy, and we recorded the corresponding testing accuracy and training speed using Falcon GPU. Additionally, the top Hessian eigenvalue, trace, median, mean, and standard deviation are all utilized to evaluate the sharpness of the loss landscape for each sharpness-based optimizer, where smaller values of these metrics (closer to 0) indicate a flatter loss landscape.

From Table 1, VGG16 demonstrates the highest generalization ability with a test accuracy of 84% on unseen data, followed by Swin Transformer at 80.5%, ResNet50 at 80.2%, and ViT at 80%. ViT shows the flattest loss landscape with the lowest Hessian eigenvalue components, and ResNet50 shows the sharpest loss landscape. This is consistent with previous studies as the loss function of ViT is non-convex, while that of ResNet50 is strongly convex [38]. Additionally, ViT achieves the fastest training speed, taking only 2 minutes, whereas Swin Transformer is the most computationally expensive, requiring 21 minutes.

#### 2) EXPERIMENT 2: BASELINE MEASUREMENT VS SHARPNESS-BASED OPTIMIZERS WITH RESNET50

ResNet50 [23] is a 50-layer convolutional neural network (comprising 48 convolutional layers, one MaxPool layer, and one average pool layer) that forms networks by stacking residual blocks. This experiment compares the baseline performance of ResNet50 using Adam optimizer against sharpness-based optimizers to determine their effect on generalization, training speed, and the loss landscape.

The results from Table 2 indicate that SAM, ASAM, GSAM, and WSAM all result in a flatter loss landscape compared to the baseline Adam optimizer, whereas CRSAM shows a sharper loss landscape with high Hessian eigenvalue components. SAM, ASAM, and GSAM demonstrate higher generalization ability, with test accuracies higher than the baseline accuracy (80.2%), while WSAM and CRSAM show lower test accuracies. ASAM and GSAM both achieve the highest generalization ability with a test accuracy of 84.2%, followed closely by SAM with a test accuracy of 84.0%. However, SAM exhibits the fastest training speed at 38 minutes, followed by ASAM at 420 minutes and GSAM at 1440 minutes.

#### 3) EXPERIMENT 3: BASELINE MEASUREMENT VS SHARPNESS-BASED OPTIMIZERS WITH VGG16

VGG16 [24] is a deep convolutional neural network known for its simplicity and uniform architecture with just 16 layers (13 convolutional layers and 3 fully connected layers). This experiment compares the baseline performance of VGG16 using Adam optimizer against sharpness-based optimizers.

The results in Table 3 show that SAM, ASAM, WSAM and GSAM result in a flatter loss landscape than the baseline Adam optimizer, while CR-SAM results in a sharper loss landscape, which is consistent with our findings in Experiment 2. Also consistent with Experiment 2, ASAM and SAM exhibit the highest generalization ability with test accuracies of 89.2% and 87.5%, respectively. CR-SAM also demonstrates better generalization ability than the baseline, with a test accuracy of 86.3%, while WSAM shows equal generalization ability at 84%, and GSAM exhibits the worst generalization ability at 78.2%. SAM and CR-SAM are significantly faster than the other sharpness-based optimizers, with training speeds of 24 minutes and 16 minutes, respectively, while ASAM is the third fastest with a training speed of 840 minutes.

Experiments 2 and 3 indicate that SAM and ASAM have the best overall performance compared to the Adam optimizer and other flatness methods when using ResNet50 and VGG16.

#### 4) EXPERIMENT 4: BASELINE MEASUREMENT VS SHARPNESS-BASED OPTIMIZERS WITH VIT

Vision Transformers (ViT) [25] is an architecture that uses self-attention mechanisms to process images using transformer blocks, where each block consists of two sub-layers; a multi-head self-attention layer and a feed-forward layer. This experiment compares the baseline performance of ViT using Adam optimizer against sharpness-based optimizers to evaluate if they cause any effect on generalization, training speed and the loss landscape.

The results from Table 4 show that SAM, GSAM, and CRSAM result in a flatter loss landscape than the regular Adam optimizer, as evidenced by their lower Hessian eigenvalue components, while ASAM and WSAM show a sharper loss landscape. However, the only sharpness-based optimizer that shows higher generalization ability is SAM as it results in 82.5% test accuracy, while the rest result in a test accuracy less than the baseline accuracy (80%). SAM also shows high training speed with just 4 minutes.

#### 5) EXPERIMENT 5: BASELINE MEASUREMENT VS SHARPNESS-BASED OPTIMIZERS WITH SWIN TRANSFORMER

Swin Transformer [26] is a hierarchical visual transformer with an efficient shift-window partitioning scheme for computing self-attention. In this experiment, we compare the baseline performance of the Swin Transformer using the Adam optimizer to that of the sharpness-based optimizers. We evaluate generalization, training speed, and the geometry of the loss landscape.

The results from Table 5 show that only SAM and CR-SAM result in a flatter loss landscape compared to the baseline Adam optimizer, as indicated by their lower Hessian eigenvalue components, while the other methods exhibit a sharper loss landscape. Consistent with the results in Experiment 4, only SAM demonstrates higher generalization performance than the baseline Adam optimizer, with a test accuracy of 82.3%, whereas the other sharpness-based optimizers result in test accuracies below 80.5%.

#### 6) EXPERIMENT 6: CROSS-DOMAIN EVALUATION OF BASELINE MEASUREMENT VS SHARPNESS-BASED OPTIMIZERS

To assess the generalization capability of sharpness-based optimizers, we conducted a cross-domain evaluation using the BUSIS dataset [35], which consists of 562 breast ultrasound images (306 benign, 256 malignant). In this experiment, models were trained on the GDPH&SYSUCC dataset (used in previous experiments) and tested on BUSIS, ensuring no exposure to the target domain during training.

We compared the Adam optimizer with sharpness-aware methods, including SAM, ASAM, GSAM, WSAM, and CR-SAM, across four model architectures: ResNet50, VGG16, ViT, and Swin Transformer. Hyperparameter settings were kept consistent with prior experiments to ensure fair comparisons.

Table 6 presents the cross-domain performance of each optimizer. SAM consistently improved generalization across all architectures, outperforming Adam and other sharpness-based optimizers. ASAM, GSAM, WSAM, and CR-SAM exhibited mixed performance, with ASAM showing strong results in CNNs but poor performance in ViT and Swin Transformer. These findings suggest that while SAM enhances robustness across domains, other sharpness-based methods may require further tuning for optimal cross-domain generalization.

#### 7) EXPERIMENT 7: GENERALIZATION PERFORMANCE OF BASELINE MEASUREMENT VS SHARPNESS-BASED OPTIMIZERS ON CHEST X-RAY

To further assess the generalization performance of sharpness-based optimizers, we conducted an experiment on chest X-ray classification using the CheXpert dataset [39], a large-scale dataset for automated chest radiograph interpretation. CheXpert contains 224,316 chest radiographs from 65,240 patients, with labels for 14 different lung diseases and conditions. The dataset includes both frontal and lateral chest radiographs; however, there are only 33,087 lateral radiographs compared to 191,229 frontal radiographs. To ensure uniformity, all lateral radiographs were removed, following established preprocessing protocols in comparable studies [40]. The original CheXpert partition provides separate training and validation sets. However, to ensure statistically robust evaluation and mitigate potential biases from the relatively small validation set, we adopted an 80/20 train-test split after combining all available data.

We trained models on the CheXpert training set and evaluated their performance on the CheXpert test set to assess within-domain generalization. We tested four model architectures: ResNet50, VGG16, ViT, and Swin Transformer, using the standard Adam optimizer as the baseline and comparing it against sharpness-aware optimizers, including SAM, ASAM, GSAM, WSAM, and CR-SAM.

The results in Table 7 indicate that sharpness-aware optimization methods generally improved generalization performance on the CheXpert dataset compared to the baseline Adam optimizer. Among the tested methods, ASAM achieved the highest accuracy for ResNet50 (80.1%) and VGG16 (79.1%), while SAM consistently improved performance across all architectures. GSAM also showed competitive results, particularly for ResNet50 (79.6%). However, performance gains were less pronounced for ViT and Swin Transformer, with ASAM and GSAM showing declines compared to Adam in these architectures. These results suggest that sharpness-aware optimization can enhance generalization in convolutional models but may require further tuning for transformer-based architectures.

#### 8) EXPERIMENT 8: GENERALIZATION PERFORMANCE AND COMPUTATIONAL ANALYSIS OF BASELINE MEASUREMENT VS SHARPNESS-BASED OPTIMIZERS ON COLON CANCER HISTOPATHOLOGY

To further expand the scope of our evaluation, we assessed both the generalization performance and computational overhead of sharpness-based optimizers on PathMNIST, a colon cancer histopathology dataset from MedMNIST [41]. PathMNIST comprises 107,180 hematoxylin and eosin (H&E)-stained tissue patches classified into nine categories. Following the standard dataset split, we allocated 89,996 images for training, 10,004 for validation, and 7,180 for testing.

For this experiment, we also tested four model architectures: ResNet50, VGG16, ViT, and Swin Transformer, using the standard Adam optimizer as the baseline and comparing it against sharpness-aware optimizers, including SAM, ASAM, GSAM, WSAM, and CR-SAM. Furthermore, we compared the computational efficiency of SAM and its variants against Adam, with a relative speed of 1.0, to evaluate the additional computational overhead introduced by the sharpness-based optimizers.

The results in Table 8 indicate that sharpness-aware optimization methods generally improved generalization performance on the PathMNIST dataset compared to the baseline Adam optimizer. For all models, SAM outperformed the Adam baseline in accuracy, with the largest improvements seen in ResNet50 (88.4% vs. 86.1%) and ViT (82.5% vs. 78.9%). Notably, SAM’s computational speed, with a factor of 1.90 for ResNet50 and 1.83 for VGG16, indicates a reasonable trade-off between performance and efficiency. In contrast, while ASAM and GSAM offered the highest accuracy improvements, especially for ResNet50, they introduced considerable computational overhead, with speed factors exceeding 4.5. Thus, SAM provides a balanced trade-off between accuracy and speed.

To further analyze the effect of sharpness-based optimizers on model performance, we analyzed the geometry of the loss landscapes of the ResNet50 model trained on the PathMNIST dataset. The loss landscape provides valuable insights into the optimization process by illustrating the geometry of the loss surface, helping to understand the sharpness and stability of the minima found by different optimizers. By comparing the loss landscapes of the baseline Adam optimizer and sharpness-based optimizers such as SAM, ASAM, GSAM, WSAM, and CRSAM, we aim to explore how each optimizer affects the shape and smoothness of the loss surface. Following the visualization techniques from Li et al. [42], the loss values were visualized along two orthogonal Gaussian perturbation directions sampled around the local minima.

**FIGURE 2.**
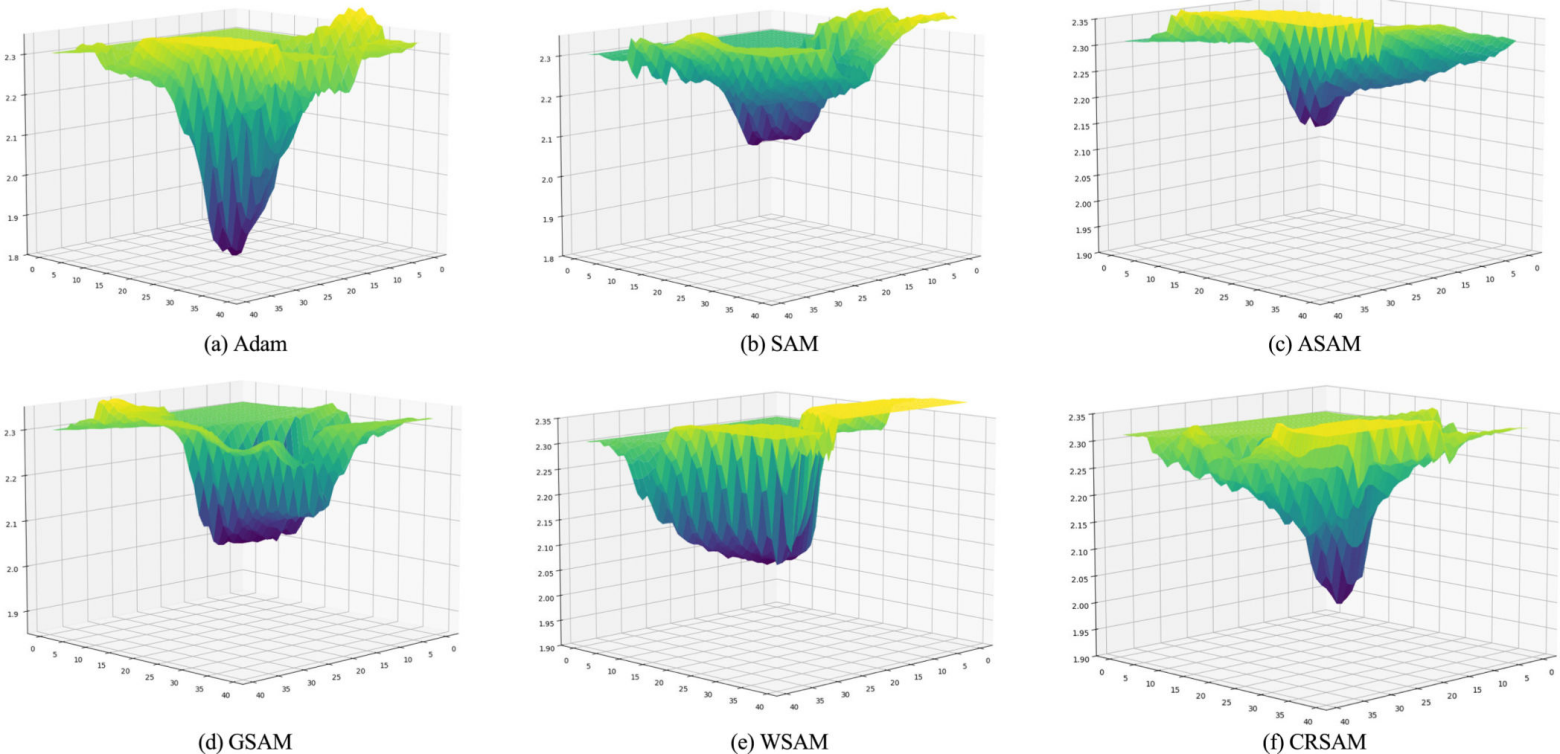
Comparisons of loss landscapes for Adam, SAM, ASAM, GSAM, WSAM, and CRSAM on the PathMNIST dataset using ResNet50 model.

The loss landscape visualizations, shown in Figure 2, validate the Hessian eigenvalues reported in Table 8, further illustrating the effectiveness of sharpness-based optimizers in flattening the loss surface. Specifically, SAM and ASAM resulted in significantly lower Hessian eigenvalues, indicating smoother and more stable minima compared to the baseline Adam optimizer.

## IV. DISCUSSION

**FIGURE 3.**
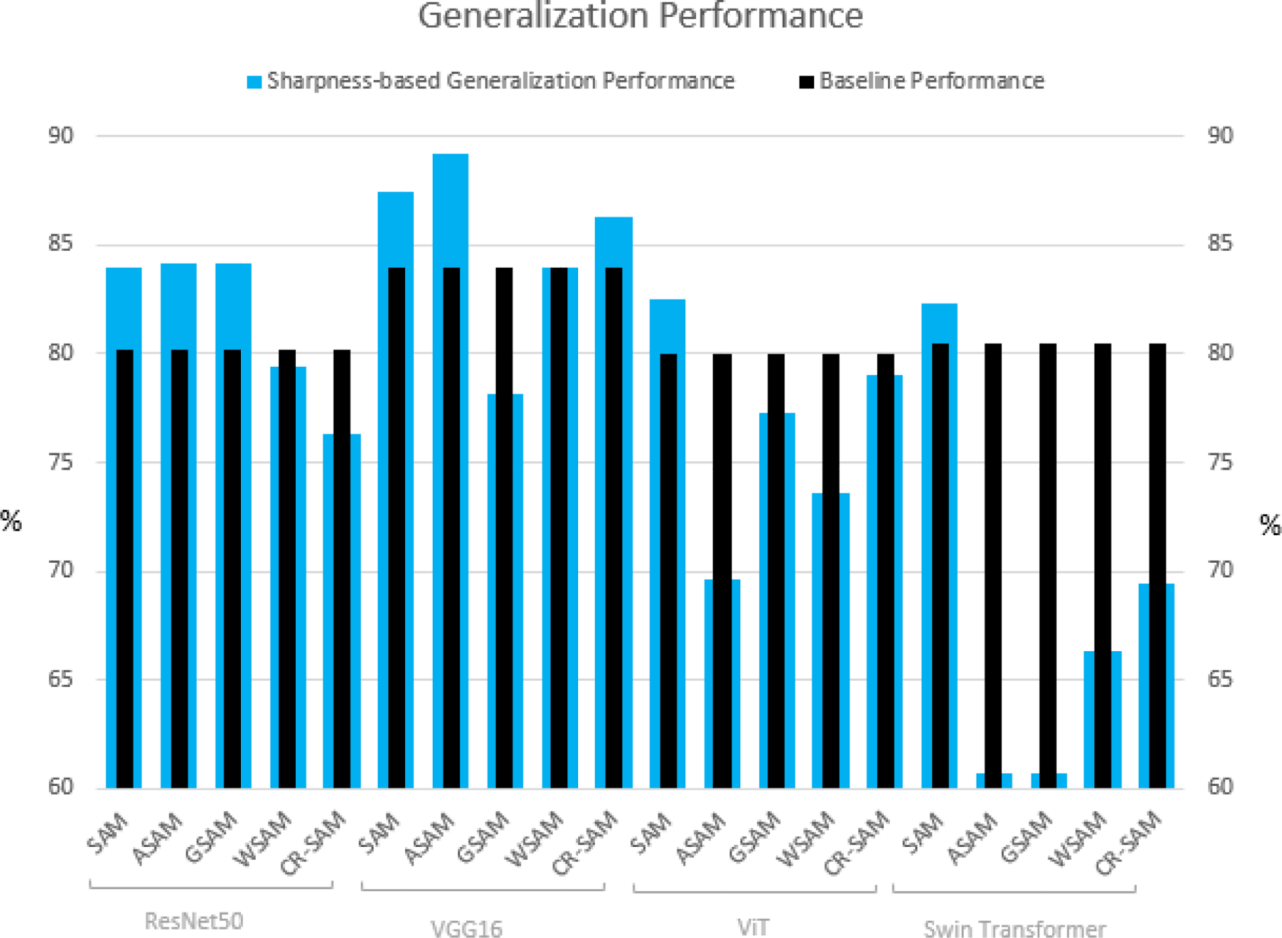
Generalization performance of sharpness-based optimizers against the best baseline performance of Adam optimizers.

In this work, we evaluate the generalization performance of the most common sharpness-based optimizers for a medical image classification task, comparing them to the best baseline generalization performance achieved with the Adam optimizer. Toward this goal, we conducted experiments using two CNN-based classification models (ResNet50 and VGG16) and two Vision Transformer models (ViT and Swin Transformer). Our results show, as illustrated in Figure 3, that SAM consistently achieved higher generalization performance than the Adam optimizer across all tested models. ASAM demonstrated improved generalization performance for CNN-based models but performed poorly for Vision Transformers. Additionally, GSAM improved generalization performance only for ResNet50, while CR-SAM showed improvements only for VGG-16. WSAM, however, failed to enhance generalization performance for any of the tested models.

While several sharpness-based optimizers improved generalization, they varied significantly in computational efficiency. Among them, SAM offered the most balanced trade-off between performance and cost, making it suitable for real-world applications. CR-SAM also demonstrated strong computational efficiency, achieving performance gains with minimal training overhead. In contrast, methods like ASAM, GSAM, and WSAM, while occasionally boosting accuracy, introduced higher computational demands, which may limit their scalability in time-sensitive or resource-constrained deployment scenarios. Practical deployment of sharpness-based optimizers in clinical settings requires careful consideration of real-world constraints. These include inference latency, GPU memory usage, and compatibility with low-power edge devices. Among the tested models, SAM offers the most promising balance, achieving strong generalization performance with minimal additional cost over Adam. Models like ASAM, GSAM, and WSAM may be infeasible in real-time or embedded settings due to their substantial computational overhead. Furthermore, regulatory approval and explainability remain key barriers to clinical adoption, especially for complex optimization methods that introduce additional model dynamics.

To further validate our findings, we extended our evaluation by incorporating cross-domain generalization using the BUSIS dataset, along with two large open-access medical datasets, CheXpert and PathMNIST. The results from these additional datasets align with our observations on BUS dataset, confirming that SAM remains the most effective sharpness-based optimizer for enhancing generalization performance in medical image classification. Moreover, Hessian calculations and the loss landscape visualizations, shown in Figure 2, reinforce our conclusions by demonstrating that SAM produces consistently flatter minima than the standard Adam optimizer, supporting the hypothesis that flatter loss surfaces contribute to better generalization.

The consistent advantage of SAM can be attributed to its PAC-Bayesian optimization framework, which explicitly minimizes both loss and sharpness through adversarial weight perturbation. In medical imaging tasks, where datasets often exhibit low signal-to-noise ratios and heterogeneous feature distributions, SAM’s perturbation radius (ρ) acts as an implicit regularizer by adjusting model parameters in the worst-case direction before computing the gradient update. This process ensures that the model converges to flatter minima, which is associated with improved generalization. While ASAM extends SAM by employing an adaptive perturbation norm, its looser control over sharpness reduction can lead to instability, particularly in Vision Transformers. ASAM’s normalization relies on a scaling-invariant approach, but in architectures like ViTs, where layer-wise weight scales exhibit high variance due to the self-attention mechanism, this normalization can lead to inconsistent perturbation magnitudes. These fluctuations destabilize gradient updates and impair convergence to flatter minima. This likely explains why ASAM performs well for CNNs, which tend to have more uniform scaling, but underperforms on Vision Transformers. GSAM’s surrogate gap minimization underperforms SAM because its local curvature Hessian approximation is sensitive to medical imaging noise. Additionally, its aggressive gradient orthogonalization can over-smooth decision boundaries, reducing sensitivity to subtle pathological features compared to SAM’s more balanced approach. WSAM’s underperformance stems from its reliance on a weighted sharpness term controlled by the hyperparameter γ, which can lead to suboptimal optimization, especially when the weight on sharpness is too high. This causes the model to converge to flatter minima with potentially larger loss values. Additionally, the decoupling of the regularization term from the base optimizer may hinder the model’s ability to effectively incorporate sharpness information, resulting in less efficient convergence compared to SAM’s more straightforward sharpness-focused approach. CR-SAM underperformed because its integration of the curvature regularization term based on the normalized Hessian trace, which can negatively impact generalization by over-penalizing sharp minima. Furthermore, the approximation of the Hessian trace using finite differences may introduce inaccuracies, reducing the effectiveness of the method.

In future work, we aim to further analyze the geometric properties of the loss landscape and investigate potential improvements to sharpness-based optimizers. Additionally, we plan to explore the combination of SAM with other regularization techniques to enhance generalization performance in medical image analysis. Additionally, while our evaluation spans ultrasound, X-ray, and histopathology images, the absence of modalities like MRI and CT is a limitation. Including 3D volumetric data such as BraTS (MRI) or ACDC (cardiac MR) in future work would offer a more complete understanding of sharpness-based optimizer performance across diverse medical imaging modalities. Furthermore, recent developments in large language models have explored the distillation of structured rationales from large to smaller models to improve interpretability [43]. Integrating similar rationale-based techniques into sharpness-aware training, particularly in multimodal clinical systems (e.g., combining radiology images with report generation), represents a promising future direction for improving transparency and robustness.

## V. CONCLUSION

This paper provides a comparative analysis on the most common sharpness-based algorithms for improving the generalization of neural networks. To the best of our knowledge, this paper is the first to evaluate sharpness-based optimizers for their usability in medical image analysis, specifically for CNN-based classification models and Vision Transformers. Our results indicate that Sharpness-Aware Minimization (SAM) is the only method that consistently enhances the generalization ability of all tested models. Performance comparisons based on Hessian calculations and loss landscape visualizations demonstrate that the loss landscape produced by SAM is flatter than that resulting from the standard Adam optimizer for all tested models. In contrast, ASAM, GSAM, WSAM, and CR-SAM did not exhibit consistent improvements across our experiments. These findings suggest that more research is necessary to refine SAM and its variants to further enhance generalization in medical image analysis.

## Figures and Tables

**TABLE 1.** Baseline performance ResNet50, VGG16, ViT, and Swin transformer models using adam optimizer.

Model	Test Accuracy (%)	Training time (min)	Top Hessian Eigenvalue	Hessian Median	Hessian Mean	Hessian SD	Hessian Trace
**ResNet50**	80.2	12	10631.19	17088.91	31078.76	34360.13	18202.65
**VGG16**	**84**	14	32.69	98.06	152.91	**168.26**	80.26
**ViT**	80	**2**	**19.42**	**21.47**	**127.58**	390.50	**36.14**
**Swin**	80.5	21	206.82	130.64	136.59	341.54	466.98

**TABLE 2.** Baseline performance of ResNet50 vs the most common sharpness-based optimizers.

Optimizer	Test Accuracy (%)	Training time (min)	Top Hessian Eigenvalue	Hessian Median	Hessian Mean	Hessian SD	Hessian Trace
**Adam**	80.2	12	10631.19	17088.91	31078.76	34360.13	18202.65
**SAM**	**84**	38	384.33	230.23	463.76	692.36	712.93
**ASAM**	**84.2**	420	**7.46**	**7.87**	**8.67**	**3.41**	**34.08**
**GSAM**	**84.2**	1440	260.52	88.84	286.27	654.28	314.97
**WSAM**	79.4	1450	1508.93	214.96	2419.83	5865.05	1651.63
**CRSAM**	76.3	**22**	16695.02	13893.18	15517.05	8345.49	27176.58

**TABLE 3.** Baseline performance of VGG16 vs the most common sharpness-based optimizers.

Optimizer	Test Accuracy (%)	Training time (min)	Top Hessian Eigenvalue	Hessian Median	Hessian Mean	Hessian SD	Hessian Trace
**Adam**	84	14	32.69	98.06	152.91	168.26	80.26
**SAM**	**87.5**	24	**0.01**	**0.03**	**0.47**	1.46	**0.02**
**ASAM**	**89.2**	840	5.32	5.24	5.55	**0.92**	18.46
**GSAM**	78.2	1200	19.49	19.67	9.57	21.29	−192.55
**WSAM**	84	1460	86.42	93.91	98.94	38.01	65.32
**CRSAM**	**86.3**	**16**	3935.38	478.29	245.04	982.65	3790.39

**TABLE 4.** Baseline performance of ViT vs the most common sharpness-based optimizers.

Optimizer	Test Accuracy (%)	Training time (min)	Top Hessian Eigenvalue	Hessian Median	Hessian Mean	Hessian SD	Hessian Trace
**Adam**	80	2	19.42	21.47	127.58	390.50	36.14
**SAM**	**82.5**	4	**0.65**	**12.48**	**11.08**	11.37	4.83
**ASAM**	69.6	53	82.90	40.47	43.69	39.36	1104.52
**GSAM**	77.3	56	17.49	**12.48**	12.26	11.37	−165.05
**WSAM**	73.6	32	19.38	22.67	22.70	**5.94**	5.87
**CRSAM**	79	**3**	0.79	16.77	271.53	646.09	**2.84**

**TABLE 5.** Baseline performance of Swin Transformer vs the most common sharpness-based optimizers.

Optimizer	Test Accuracy (%)	Training time (min)	Top Hessian Eigenvalue	Hessian Median	Hessian Mean	Hessian SD	Hessian Trace
**Adam**	80.5	21	206.82	130.64	136.59	341.54	466.98
**SAM**	**82.3**	48	**18.83**	**17.27**	**20.31**	88.63	**93.13**
**ASAM**	60.7	650	359.04	359.73	359.72	0.83	440.36
**GSAM**	60.7	720	300.37	300.39	300.37	**0.07**	4196.10
**WSAM**	66.3	380	680.60	684.70	685.27	27.19	1729.92
**CRSAM**	69.4	**45**	135.03	143.45	143.46	38.58	111.71

**TABLE 6.** Cross-domain generalization performance of baseline Adam optimizer vs the most common sharpness-based optimizers on BUSIS dataset.

Optimizer	ResNet50	VGG16	ViT	Swin Transformer
**Adam**	85.8	86.3	84.5	84.1
**SAM**	**86.7**	**87.5**	**85.3**	**84.4**
**ASAM**	**86.9**	**87.7**	76.6	71.3
**GSAM**	**86.2**	84.2	82.2	70.5
**WSAM**	84.6	86.1	81.7	73.6
**CRSAM**	82.9	**86.8**	83.9	76.8

**TABLE 7.** Generalization performance of baseline Adam optimizer vs the most common sharpness-based optimizers on the CheXpert chest X-ray dataset.

Optimizer	ResNet50	VGG16	ViT	Swin Transformer
**Adam**	78.2	77.5	74.6	72.5
**SAM**	**79.4**	**78.3**	**75.9**	**73.8**
**ASAM**	**80.1**	**79.1**	71.3	69.4
**GSAM**	**79.6**	77.4	72.7	67.3
**WSAM**	77.5	**77.6**	71.9	69.2
**CRSAM**	76.6	**77.9**	73.8	70.8

**TABLE 8.** Generalization performance (Test Accuracy), computational cost, and top hessian eigenvalue of baseline Adam optimizer vs the most common sharpness-based optimizers on PathMNIST dataset.

Optimizers	Accuracy	Speed	Hessian
**Adam**	86.1	1.00	10023.51
**SAM**	**88.4**	2.13	120.33
**ASAM**	**88.7**	4.51	**99.46**
**GSAM**	**87.9**	5.61	1975.79
**WSAM**	**86.3**	5.54	2142.28
**CRSAM**	85.2	**1.68**	9507.17
**(a) ResNet50**			

Optimizers	Accuracy	Speed	Hessian
**Adam**	95.4	1.00	58.50
**SAM**	**96.0**	1.90	**8.36**
**ASAM**	**96.4**	4.73	10.48
**GSAM**	93.2	5.83	69.7
**WSAM**	94.9	5.61	62.53
**CRSAM**	**95.7**	**1.21**	75.28
**(b) VGG16**			

Optimizers	Accuracy	Speed	Hessian
**Adam**	86.7	1.00	29.15
**SAM**	**87.1**	1.83	**5.21**
**ASAM**	79.2	4.21	90.23
**GSAM**	85.1	4.84	81.24
**WSAM**	83.5	4.72	45.18
**CRSAM**	84.8	**1.31**	25.1
**(c) ViT**			

Optimizers	Accuracy	Speed	Hessian
**Adam**	78.9	1.00	316.24
**SAM**	**82.5**	**2.04**	**29.4**
**ASAM**	77.2	4.43	451.24
**GSAM**	73.9	5.89	604.74
**WSAM**	76.2	5.72	542.19
**CRSAM**	78.6	**1.87**	295.37
**(d) Swin Transformer**			
